# Can Physicians Delay Appendectomy for One Night in Children With Acute Appendicitis?

**DOI:** 10.34172/mejdd.2024.369

**Published:** 2024-01-31

**Authors:** Amrollah Salimi, Seyed Mojtaba Alavi, Mojdeh Bahadorzadeh, Mostafa Vahedian, Enayatollah Noori, Gulnaz Rezaie

**Affiliations:** ^1^Department of Surgery, School of Medicine, Shahid Beheshti Hospital, Qom University of Medical Sciences, Qom, Iran; ^2^General Practitioner, Qom University of Medical Sciences, Qom, Iran; ^3^Department of Epidemiology and Biostatistics, Research Center for Environmental Pollutants, Qom University of Medical Sciences, Qom, Iran

**Keywords:** Acute appendicitis, Children, Appendectomy

## Abstract

**Background:**

In pediatrics, appendicitis is the leading cause of emergency surgery. It was previously believed that postponing the surgery could lead to the appendix rupture. Children with this condition can be difficult to diagnose. The evidence regarding the necessity of an immediate appendectomy is a topic of debate. In this study, we evaluated the medical records of patients who were diagnosed with acute appendicitis to determine whether postponing appendectomy for one night is safe or not.

**Methods:**

This study involved 534 individuals diagnosed with acute appendicitis, who were separated into two groups: those who underwent an appendectomy immediately (within 8 hours) and those who had a delayed procedure (between 8-18 hours). We recorded and compared demographic data, symptoms, laboratory results, time of symptoms, hospitalization duration, surgery duration, overall time, length of stay after surgery, and any other complications that occurred between the two groups.

**Results:**

The rate of surgical site infection (SSI) did not differ significantly between the groups (2.8% vs 4.2%, *P*=0.74). Additionally, there was no significant difference in the risk of perforation between the time of surgery in our study (21.9% vs 19.8%, *P*>0.05).

**Conclusion:**

Our findings suggest that there is no increased risk of complications such as perforation when appendectomy is delayed for up to 18 hours.

## Introduction

 Appendicitis is known as the most common cause of emergent pediatric surgery. It is estimated that around 80 000 appendectomies are performed on children in the United States each year.^1,2^ Perforation, abscess formation, peritonitis, and bowel obstruction are some of the major complications of acute appendicitis.^3^ It was believed that a delay in appendectomy would increase the risk of perforated appendicitis. Therefore, emergency appendectomy was chosen as the standard treatment for acute appendicitis.^4^ The necessity of emergent appendectomy is controversial. Some studies suggest that emergent surgery is needed to reduce appendicitis complications such as perforation, whereas some other studies have demonstrated that a 24-hour delay does not increase the risk of complications and perforation.^5-12^ Few studies have indicated that appendectomies delayed for more than 48 hours are associated with a higher risk of complications and perforation.^6,7,12^ It is worth noting that some studies suggest that the process of perforated appendicitis may not necessarily be a time-dependent phenomenon.^13^ Acute appendicitis is a common diagnosis in children presenting with abdominal pain. In-hospital delays may contribute to the incorrect diagnosis of acute appendicitis in pediatrics because the diagnosis of this disease in children can be challenging at times. Additionally, some hospitals may not have a surgical team available during night-time hours. In this study, we investigated whether it would be safe or not to delay the appendectomy until the following day, after completing the work-up with a definitive diagnosis and a fresh surgical team.

## Materials and Methods

 This retrospective study investigated children who underwent surgical diagnosis for acute appendicitis. The study included patients referred to hospitals affiliated with Qom University of Medical Sciences between March 21, 2012, and March 21, 2021. The protocol of the study was approved by the Research Ethics Committees of Qom University of Medical Sciences (IR.MUQ.REC.1401.027). We reviewed the medical records of 550 patients who underwent surgery for acute appendicitis in two hospitals. Some surgeries were performed in a teaching hospital where operation rooms were available whenever needed. Initially, patients presented to the emergency department (ED), where primary evaluations and examinations were performed by ED physicians. After a diagnosis of acute appendicitis, a surgical consult was requested. Most of the patients underwent open appendectomy, and all of them received antibiotics before surgery. The individuals were divided into two groups: immediate appendectomies ( < 8 hours) and delayed appendectomies (8-18 hours). The overall time was from the time of ED registration until the time of surgery. Demographic information including age, sex, underlying diseases, and other factors such as signs and symptoms, laboratory data, symptomatic time, time of admission, time of surgery, overall time, length of postoperative stay, surgical site infection (SSI), abscess formation, perforation, sepsis, peritonitis, and mortality were captured by reviewing medical records. To investigate whether complications of appendicitis are related to the clinical progress of patients, we used regression models to estimate the association between the dependent variable of complications and the independent variable of clinical progress. The continuous variables were analyzed using a dependent t-test, followed by a Mann-Whitney U test for non-normally distributed data. The categorical variables were analyzed using a chi-square test. A *P* value of less than 0.05 was considered statistically significant. All data analysis was performed using IBM® SPSS® software for Windows® version 26.

## Results

 534 patients who underwent an appendectomy at the Qom University of Medical Science hospitals between March 2012 and March 2021 were enrolled in this study. 16 patients were excluded from the study due to underlying diseases and non-appendicitis diagnoses. 54.5% of the patients were male. 40.3% of individuals (221) underwent surgery within 8-18 hours (delayed appendectomy). The mean age ± SD of the two groups was 7 ± 3.2 and 7 ± 3.1 in < 8 h and > 8 h groups, respectively. The presence of underlying diseases was rare. Each group did not have significant differences in clinical symptoms and signs.

 All individuals underwent an open appendectomy. The rate of SSI was 2.8% and 4.2% among the immediate and delayed groups, respectively. There was no correlation between the time of appendectomy and the increased risk of SSI (*P* = 0.74). In the immediate group, 7.5% of patients developed peritonitis compared with 2.8% in the delayed group (*P* = 0.019). The overall perforation rate was 19.8% (n = 106). Of those who underwent appendectomy within 8 hours, 21.9% had perforated appendicitis, while the perforation rate was 16.7% in delayed appendectomies. There was no significant difference between the time of surgery and the increased risk of perforation in our study (*P* > 0.05). A delayed appendectomy was not associated with a longer postoperative stay (*P* > 0.05, Table 1).

**Table 1:** Length of postoperative stay in each group of patients

	**2 Days**	**3 Days**	**4days**	**5 Days**	**6 Days**	**7 Days**
Immediate group ( < 8 hours)	187	78	38	13	2	1
	58.6%	24.5%	11.9%	4.1%	0.6%	0.3%
Delayed group (8-18 hours)	132	50	18	12	2	1
	61.4%	23.3%	8.4%	5.6%	0.9%	0.5%

## Discussion

 Children may present with unusual clinical and laboratory findings, thus, the diagnosis of acute appendicitis may be challenging and delayed in children.^14^ The impact of a postponed appendectomy on the augmented possibility of perforation in 857 children was assessed by Meltzer and colleagues.^15^ They showed that the risk of perforation increased by 2% for every hour of delay between ED triage time and appendectomy.^15^ Patients who experienced a delayed diagnosis of appendicitis during the COVID-19 lockdown had a higher occurrence of complicated appendicitis, including peritonitis and peritoneal abscesses, according to a study.^16^ On the other hand, van Dijk and colleagues proposed that doctors could postpone the appendectomy for a maximum of 24 hours in the absence of any indications for complex appendicitis.^17^ According to their report, the delay in treatment for appendicitis does not pose a higher risk of complications such as SSIs and morbidity. In another study conducted by Abdul Jawad and colleagues, it was found that a delay of more than 24 hours did not increase the likelihood of complicated appendicitis.^18^ The overall perforation rate in our study was 19.8%, which was similar to a previous study.^7^ Some studies suggest that in-hospital delay increases the risk of appendiceal perforation, while other studies claim that in-hospital delay of up to 24 hours does not increase the rate of perforation.^17,19^ The present research discovered that cases with perforation experienced lesser in-hospital delay time, with no notable variation between the two groups. Despite our results indicating that postponing appendectomy for up to 18 hours does not increase the chances of SSI, certain authors propose that delaying the procedure within the hospital can indeed augment the risk of SSI, even if it is only for 6-12 hours.^20^

 In this study, patients who developed peritonitis were more in the immediate group. It may be due to their severe presentation at the ED and prompt surgery. Aiken and colleagues reported that patients who underwent appendectomy with a delay of 12-24 hours did not have higher postoperative complications, except for a longer postoperative hospital stay.^21^ They suggested prompt appendectomy for patients to lower hospital costs. Other authors have concluded that appendectomies should not be excessively delayed because acute appendicitis may progress to complicated appendicitis, resulting in a longer length of stay (LOS).^22^ Our data show that delayed appendectomy does not increase LOS. The reason for this may be our shorter delay of appendectomy (up to 18 hours) compared with other studies. However, our study has some limitations. Firstly, our patients underwent surgery within 18 hours, which is a shorter delay compared with other studies that showed a delay of more than 48 hours was a risk factor for appendicitis complications such as appendicular perforation.^7,23^ Secondly, all of our participants were from a single center. Further multicenter studies may yield more accurate findings. Thirdly, all of our patients underwent open appendectomies, while patients in other studies underwent laparoscopic appendectomies.^21^

## Conclusion

 Our findings show that a one-night delay does not increase the risk of complications related to appendicitis, such as perforation, LOS, and SSI. Moreover, it allows surgeons to conduct further investigations, such as spiral abdominal computed tomography. Additionally, not all hospitals have operating rooms available during night-time hours. Overall, this situation may vary between centers depending on factors such as hospital facilities and availability of the surgical team and operation rooms.
